# Immunoprofiles associated with controlled human malaria infection and naturally acquired immunity identify a shared IgA pre-erythrocytic immunoproteome

**DOI:** 10.1038/s41541-021-00363-y

**Published:** 2021-09-13

**Authors:** Andrea A. Berry, Joshua M. Obiero, Mark A. Travassos, Amed Ouattara, Drissa Coulibaly, Matthew Adams, Rafael Ramiro de Assis, Aarti Jain, Omid Taghavian, Andrew Sy, Rie Nakajima, Algis Jasinskas, Matthew B. Laurens, Shannon Takala-Harrison, Bourema Kouriba, Abdoulaye K. Kone, Ogobara K. Doumbo, B. Kim Lee Sim, Stephen L. Hoffman, Christopher V. Plowe, Mahamadou A. Thera, Philip L. Felgner, Kirsten E. Lyke

**Affiliations:** 1 Center for Vaccine Development and Global Health, University of Maryland School of Medicine, Baltimore, MD USA; 2 Department of Physiology and Biophysics, School of Medicine, University of California, Irvine, CA USA; 3 Malaria Research and Training Center, University of Sciences, Techniques, and Technologies of Bamako, Bamako, Mali; 4 Sanaria Inc., Rockville, MD USA

**Keywords:** Malaria, Antibodies, Protein vaccines

## Abstract

Knowledge of the *Plasmodium falciparum* antigens that comprise the human liver stage immunoproteome is important for pre-erythrocytic vaccine development, but, compared with the erythrocytic stage immunoproteome, more challenging to classify. Previous studies of *P. falciparum* antibody responses report IgG and rarely IgA responses. We assessed IgG and IgA antibody responses in adult sera collected during two controlled human malaria infection (CHMI) studies in malaria-naïve volunteers and in 1- to 6-year-old malaria-exposed Malian children on a 251 *P. falciparum* antigen protein microarray. IgG profiles in the two CHMI groups were equivalent and differed from Malian children. IgA profiles were robust in the CHMI groups and a subset of Malian children. We describe immunoproteome differences in naïve vs. exposed individuals and report pre-erythrocytic proteins recognized by the immune system. IgA responses detected in this study expand the list of pre-erythrocytic antigens for further characterization as potential vaccine candidates.

## Introduction

Despite control efforts, malaria remains an infectious disease of formidable impact. Since 2000, global malaria mortality has decreased by about half, to 405,000 in 2018^1^. Unfortunately, progress has plateaued, and billions of people live in areas of endemic transmission with illness impeding social and economic well-being. Naturally acquired immunity to malaria builds after repeated exposure and prevents illness^2–4^; however, it does not prevent re-infection^5^. A pre-erythrocytic vaccine that attenuates or prevents liver-stage infection, blood stage parasitemia, and gametocyte transmission would be a critical tool for malaria elimination.

Blood stage parasites that can be propagated in vitro have been extensively studied, and the blood stage proteome is well characterized; however, the antigens comprising the pre-erythrocytic proteome in the human liver have been more difficult to classify^6–9^. Moreover, the pre-erythrocytic phase of *Plasmodium* parasite development represents a bottleneck during which infection is confined to the liver without symptoms or transmission and is thus an ideal target for vaccine-induced immunity. Therefore, identification of new pre-erythrocytic vaccine targets is a priority that would aid vaccine development. To accomplish this, we previously developed a rationale to classify antigens recognized by the human immune system within days after controlled human malaria infection (CHMI) and before blood stage parasitemia is detected by PCR^10,11^. The pre-erythrocytic antigens discovered with this approach are distinct from blood stage antigens recognized by naturally exposed individuals experiencing blood stage parasitemia. Residents of malaria endemic regions develop naturally acquired immunity that includes elevated antibody levels against blood stage parasites that interfere with blood-stage parasite replication and reduce parasitemia levels enough to prevent individuals from experiencing debilitating symptoms^12^. However, parasitemia in asymptomatically infected individuals with naturally acquired immunity is sufficient to sustain transmission^13^.

Several life cycle stages of *Plasmodium falciparum*, the deadliest species, occur at various sites in the human host, and are accessible to antibodies detectable in the peripheral circulation. Sporozoites, the parasite stage that invades hepatocytes, are predominantly coated with circumsporozoite protein (CSP) and other proteins. After hepatocyte invasion, merozoites are released from liver schizonts into the peripheral circulation where merozoite surface proteins elicit antibody responses, including antibodies directed against erythrocyte invasion machinery. Merozoites then invade and mature inside red blood cells, where variant surface antigens including PfEMP1 are expressed by the parasite on the surface of infected red blood cells, facilitating endothelial adhesion and sequestration in the brain^14^ and placenta^15–17^ while also eliciting antibody responses^18–20^. A minority of blood stage parasites further differentiate into gametocytes that are taken up by the mosquito to complete the lifecycle; antibodies to these gametocytes are also taken up by the mosquito and result in transmission reducing immunity, and possibly transmission enhancing immunity^21^. In all cases, the prevailing assumption is that after IgM is initially expressed by B cells, isotype switching results in an IgG predominant antibody response with observed and purported functions that promote protection from illness^22^.

By contrast, IgA, which is found in the serum and in mucosal secretions, is not thought to be induced by *P. falciparum*. Secretory IgA is a homodimer typically elicited by antigens presented to mucosal sites where known functions include inhibition of virus infectivity, bacterial toxin neutralization, and prevention of bacterial adhesion to host cells^23^. Nonetheless, IgA could hypothetically be induced in response to pre-erythrocytic antigen traversal through liver mucosal surfaces, a proposed explanation for the presence of mucosal associated invariant T (MAIT) cells enriched for CD161 in non-human primate livers following immunization with whole organism sporozoite vaccine^24^. Alternatively, infected red blood cells at the intestinal-vascular barrier may have immune effects such as induction of T-cells with gut-homing properties^25^ or perhaps IgA induction. These hypotheses support the scant previous observations of IgA antibody induction following malaria infection or vaccination^26,27^.

Here we report an expanded pre-erythrocytic immunoproteome that includes IgA reactive antigens in acutely infected CHMI volunteers who were administered infectious sporozoites either by mosquito bite or by intravenous (IV) direct inoculation. We compared the immunoproteomes from these two routes of administration to the immunoproteome resulting from infection in children living in Mali who had previous malaria exposure.

## Results

### Specimen collection

**Fig. 1:**
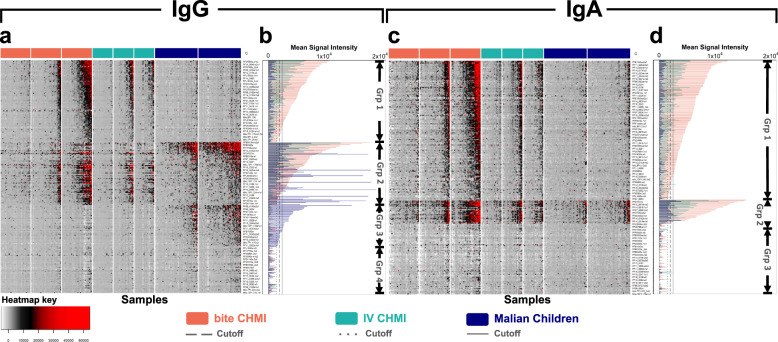
Heatmaps of *P. falciparum* antibody responses in three clinical studies. Fluorescence intensities (FI) for IgG (**a**) and IgA (**c**) (in rows; 228 and 193 antigens for IgG and IgA, respectively), and serum samples (in columns). Bar graphs for IgG (**b**) and IgA (**d**) show mean reactivity for each study cohort and antigen at day +29 for CHMI studies and the post-infection timepoint for Malian children. Cutoff lines for each cohort indicate the threshold average FI above which the cohort is considered to have reacted to that antigen. Samples were collected at baseline, day +15, and day +29 for the bite CHMI (*n* = 32); baseline, day +29, and day +57 for the intravenous (IV) CHMI (*n* = 22); and pre- and post- infection for Malian children (*n* = 47). Each FI is the raw value minus the mean of the 7 no-DNA control spots. For each group and timepoint, samples are arranged with increasing reactivity from left to right; antigens are arranged by decreasing reactivity from top to bottom in three groups. Groups (Grp) delineate antigen reactivity among cohorts as follows: IgG (**a**, **b**) Grp 1, antigens that are reactive in CHMI participants but not in Malian children; Grp 2, antigens reactive in both CHMI participants and Malian children; Grp 3, antigens reactive in Malian children but not CHMI participants (IgG), Grp 4, antigens that were non-reactive. IgA (**c**, **d**) shows only three groups; Grp 1, reactive antigens in CHMI participants but not in Malian children; Grp 2, reactive antigens in both CHMI participants and Malian children; and Grp 3, non-reactive antigens. Heatmap key contains FI color gradient.

Protein microarrays displaying a total of 251 *P. falciparum* (Pf) polypeptides representing 214 unique Pf3D7 genes were probed with sera from three cohorts (Table 1). In the mosquito-bite CHMI^28,29^, blood samples were collected 1, 15, and 29 days post-infection; samples from 32 volunteers were used for the current study. In the intravenous (IV) CHMI^30^, blood samples were collected 1, 29, and 57 days post-infection; samples from the 22 volunteers who were parasitemic after challenge were used for the current study. In the third study, 47 Malian children ages 1–6 years of age who were control participants in a vaccine study^31^ were monitored longitudinally during the malaria transmission season beginning in May 2007 for naturally acquired infection, and pre- and post-infection blood samples were selected for the current study. Heat maps and bar graphs showing the IgG and IgA antibody responses after CHMI challenge and natural exposure are shown in Fig. 1.

**Table 1:** Clinical characteristics of included studies.

	bite CHMI	IV CHMI	Malian children
Participant susceptibility	malaria naïve adults	malaria naïve adults	malaria-exposed aged 1–6 years
Study design	Safety and infectivity of CHMI administered by 1, 3, or 5 bites of aseptically reared mosquitoes with PfSPZ in salivary glands	Safety and infectivity of 7G8 strain CHMI administered intravenously (IV) by direct venous inoculation of 800–4800 aseptic, purified, cryopreserved PfSPZ	Vaccine study of AMA1/AS02A with longitudinal follow-up; current study includes a subset of the control group (rabies vaccine)
No. of participants included of total in study	32 of 37	22 of 30	47 of 400
Parasite strains	PfNF54	Pf7G8 (*n* = 17) PfNF54 (*n* = 5)	Endemic malaria infections
Study days included	Days 1, 15, 29	Days 1, 29, 57	Pre-infection sample: 6–41 days before infection Post-infection sample: 14–42 days after infection
Diagnosis	Daily microscopy	Daily ultra-sensitive PCR	Microscopy when symptoms were present

### A shared IgA immunoproteome following CHMI and a subset of Malian children after natural infection

The IV and bite CHMI induced IgG antibody responses against a collection of antigens that were not reactive in naturally exposed children (Grp 1, Fig. 1a, b). Similarly, naturally exposed children produced IgG antibodies against antigens to which sera from CHMI volunteers did not react (Grp 3, Fig. 1a, b). A third collection of antigens was reactive in both the CHMI and naturally exposed groups (Grp 2, Fig. 1a, b). IV and bite CHMI induced robust IgA antibodies against a collection of antigens that were only weakly reactive in naturally exposed children (Grp 2, Fig. 1c, d).

**Fig. 2:**
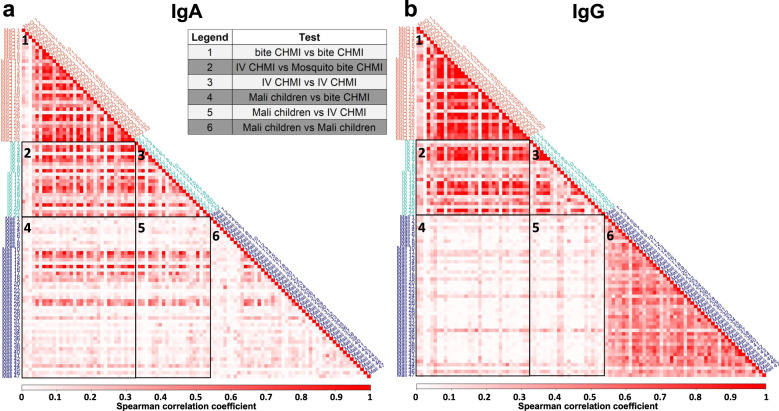
Two CHMI studies induced similar *P. falciparum* antibody profiles for IgA and IgG. IgA (**a**) and IgG (**b**) pairwise correlation matrices of the distribution of fluorescence intensity values across all proteins for each post-infection sample from three clinical studies show qualitatively different patterns of correlation. The color bar below each matrix shows the degree of correlation; the intensity of the color of the squares (light to dark red) is proportional to the value of the corresponding Spearman correlation coefficient. Samples were clustered based on the study comparison with each correlogram represented by a number as per the legend table. Samples for the Malian children were ordered by age of the participant. IgA correlation between the CHMI groups and the Malian children (more intense vs. less intense rows in panels 4 and 5) were used to subset the Malian children into a highly correlated group (*n* = 10) and a low-correlation group (*n* = 37).

Correlation matrices were created to compare profiles of Spearman correlation coefficients for the distribution of fluorescence intensity values across all proteins for each participant versus all other participants. For the CHMI cohorts and IgA (Fig. 2a panel 1, 2, and 3) and IgG (Fig. 2b panel 1, 2, and 3) analyses, most participants were highly correlated to others in the CHMI groups but not to the Malian children. However, for IgA, a subset of children from Mali had correlated profiles when compared to CHMI participants (Fig. 2a panels 4 and 5). In subsequent analyses, the children were sub-grouped into two groups that were “highly IgA correlated” (*n* = 10) and “low IgA-correlated” (*n* = 37) with the adult CHMI groups (Supplementary Fig. 1). IgA responses in Malian children had little correlation when compared against each other (Fig. 2a panel 6). For IgG, the antibody profiles for CHMI participants vs. the Malian children showed little correlation (Fig. 2b panel 4 and 5). Most Malian participants had similar IgG antibody profiles that were highly correlated to each other (Fig. 2b panel 6).

**Fig. 3:**
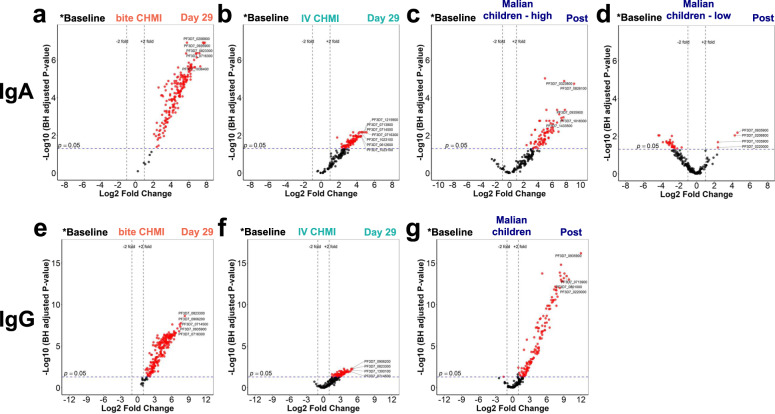
Changes in IgA and IgG antibody responses to *P. falciparum* proteins in three clinical studies. Volcano plot of IgA (**a**–**d**) and IgG (**e**–**g**) comparing baseline (CHMI timepoint day 1) signal intensities to the study-specific post-infection timepoint day 29 for bite CHMI (*n* = 32) (**a**, **e**), IV CHMI (*n* = 22) (**b**, **f**) and post-infection for Malian children (*n* = 47) (**c**, **d**, **g**), with the Malian children grouped by high vs. low correlation with the CHMI adults (as illustrated in Fig. 2). Antigens are colored red if they pass the thresholds for false discovery rate (FDR) and Log Fold change; the top antigens by *p* value and fold change are labeled. Antigens above the blue dashed line have Wilcoxon rank sum *p* < 0.05 after correction using the Benjamini Hochberg procedure. Analysis was done on 193 antigens for IgA and 228 antigens for IgG.

Volcano plots (Fig. 3) highlight antigens that displayed a significant increase in antibody response post-infection compared to baseline reactivity in the two CHMI studies. IgA antibodies to 187 antigens increased after mosquito bite CHMI (Fig. 3a) and to 91 antigens after IV CHMI (Fig. 3b); 89 IgA reactive antigens were shared between mosquito bite and IV CHMI. For the “highly correlated” Malian children (Fig. 2a panels 4 and 5), 66 antigens had increased IgA reactivity post-infection compared to malaria naïve subjects at baseline (Fig. 3c). PF3D7_0716300 (conserved Plasmodium protein) was among the top five most immunogenic antigens in both CHMI cohorts. PF3D7_0935900 (ring-exported protein 1) was among the top five most immunogenic antigens in the bite CHMI and in the highly correlated Malian children (Supplementary Table 1). PF3D7_0206800 (merozoite surface protein 2), PF3D7_0823300 (histone acetyltransferase GCN5), and PF3D7_1036400 (liver stage antigen 1) were other top-5 immunogenic antigens in the bite CHMI cohort. PF3D7_0714500 (transcription elongation factor s-II, putative), PF3D7_0713900 (conserved Plasmodium protein), PF3D7_1215900 (serpentine receptor, putative), PF3D7_0612600 (cytoplasmic tRNA 2-thiolation protein 1, putative) and PF3D7_1023100 (dynein heavy chain, putative) (two segments) were top-5 immunogenic antigens in the IV CHMI cohort. PF3D7_0826100 (E3 ubiquitin-protein ligase, putative), PF3D7_0323800 and PF3D7_1018300 conserved Plasmodium proteins, and PF3D7_1433500 (DNA topoisomerase 2) were other top-5 immunogenic antigens in the highly correlated Malian children. Of the four antigens that bound IgA in low-correlated Malian children (Fig. 3d), PF3D7_0206800 and PF3D7_0935900 were highly immunogenic in bite CHMI participants and PF3D7_0935900 was also immunogenic in the highly correlated Malian children. Fifty-two antigens had increased IgA reactivity for the two CHMI groups and the highly correlated Malian children, and all proteins with IgA reactivity in the highly correlated Malian children were also IgA-reactive in one or both of the CHMI groups (Supplementary Fig. 2). The highly correlated Malian children and the CHMI cohorts did not have a subset of antigens that had quantitatively higher seroreactivity in either group; i.e., seroreactivity to antigens was similar in both groups (Supplementary Fig. 3b). The bite CHMI group and IV CHMI groups also had similar seroreactivity when comparing the groups to each other; albeit the bite CHMI group had higher IgA seroreactivity and reactivity to more antigens than the IV CHMI group (Supplementary Figs. 2a and 4a).

The IgG response patterns also differed between the three cohorts. IgG antibodies increased for 215 antigens after bite CHMI (Fig. 3e), and 95 antigens after IV CHMI (Fig. 3f)﻿. Naturally exposed Malian children generated IgG antibodies post-infection against 132 antigens (Fig. 3g). Among the five most immunogenic antigens for IgG in the CHMI cohorts, three antigens PF3D7_0823300, PF3D7_0906200 (conserved Plasmodium protein), and PF3D7_0714500 (transcription elongation factor s-II) were common (Fig. 3e, f; Supplementary Table 2). The five most immunogenic antigens for IgG in Malian children were PF3D7_0935900, PF3D7_0220000 (liver stage antigen 3), PF3D7_0713900 and two segments of PF3D7_0801000 (Plasmodium helical interspersed subtelomeric family, subfamily c(PHISTc)) (Fig. 3g; Supplementary Table 2). IV CHMI and Malian children did not have common antigens among the five most immunogenic IgG antigens. PF3D7_0935900 was seen in both Malian children and bite CHMI (Fig. 3e, g; Supplementary Table 2). Twelve proteins were uniquely reactive in the Malian children but not the CHMI groups (Supplementary Fig. 2b). The Malian children and the CHMI groups each had a subset of proteins for which seroreactivity was high in one group but not the other (Supplementary Fig. 3c). As was the case for IgA, the bite CHMI group and IV CHMI groups also had similar IgG seroreactivity when comparing the groups to each other; and again the bite CHMI group had higher IgG seroreactivity and reactivity to more antigens than the IV CHMI group (Supplementary Figs. 2b and 4b).

Four antigens were highly immunogenic for both IgA and IgG among the cohorts (Supplementary Tables 1 and 2); Malian children had a significant increase in reactivity against PF3D7_0935900 (ring-exported protein 1), IV CHMI participants reacted highly against PF3D7_0714500 (putative transcription elongation factor s-II) and bite CHMI participants reacted against PF3D7_0823300 (histone acetyl transferase GCN5) and PF3D7_0935900. Of the 220 antigens that induced an immune reaction in the bite CHMI cohort, 182 (83%) induced both IgA and IgG reactivity. For the bite CHMI, only 5 and 33 antigens were uniquely immunogenic for IgA and IgG, respectively (Supplementary Fig. 5a). IV CHMI participants had 27 antigens that were uniquely IgA reactive, 31 that were IgG reactive and 64 antigens that were reactive for both IgA and IgG (Supplementary Fig. 5b). For Malian children, 36 antigens with IgA reactivity in the highly correlated group were also reactive for IgG. (Supplementary Fig. 5c).

### IgA and IgG seroprevalences differed between and among each study group

**Fig. 4:**
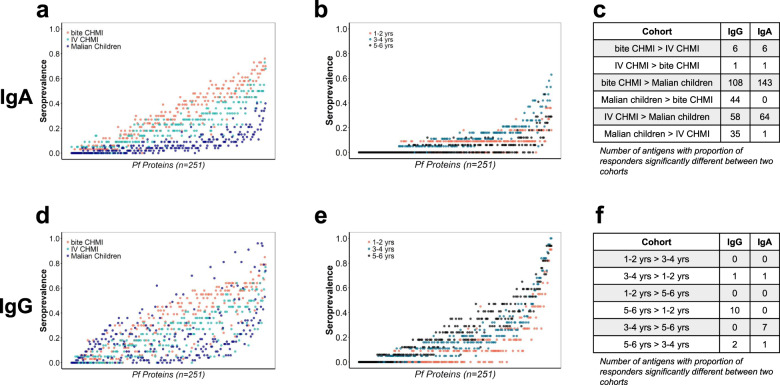
IgA and IgG seroprevalence for 251 *P. falciparum* proteins. Proteins are sorted and displayed with greatest overall seroprevalence on the right. Seroprevalence for the bite CHMI, parental CHMI, and Malian children for IgA (**a**) and IgG (**d**). Seroprevalence for three age strata of Malian children for IgA (**b**) and IgG (**e**). The tables show number of antigens with proportion of responders that are significantly different between two indicated cohorts as indicated for 193 antigens for IgA and 228 antigens for IgG (**c**, **f**), using a two-proportion *z* test with Yates continuity correction with *p* values adjusted using the Benjamini-Hochberg procedure. Data is derived from the post-infection timepoint for Malian children and day 29 for the CHMI studies.

At post-infection timepoints, 193 and 228 antigens bound IgA and IgG, respectively, in at least 10% of participants in at least one cohort (Supplementary Table 3). Proteins with a seroprevalence of <10% in each cohort were excluded from further analysis. Overall, IgA seroprevalence for individual proteins was mostly similar for the two CHMI cohorts, with only seven antigens (2.7% of antigens on the array) with significantly different proportions of responders. In contrast, both CHMI cohorts had more antigens (143 for bite CHMI and 64 for IV CHMI) with a significantly higher IgA seroprevalence than Malian children (Fig. 4a, c). Stratification of Malian children into three age groups show that for IgA, 3- to 4-year-olds had one and seven antigens with higher seroprevalence than the 1- to 2-year-olds and the 5- to 6-year-olds, respectively (Fig. 4b, f). For IgG, 5- to 6-year-olds had ten and two antigens with higher seroprevalence compared to the 1- to 2-year-olds and 3- to 4-year-olds, respectively (Fig. 4e, f).

### Number of proteins recognized by each individual (breadth score) increased after infection for IgG in all study groups and for IgA in the CHMI groups and a subset of the Malian children

**Fig. 5:**
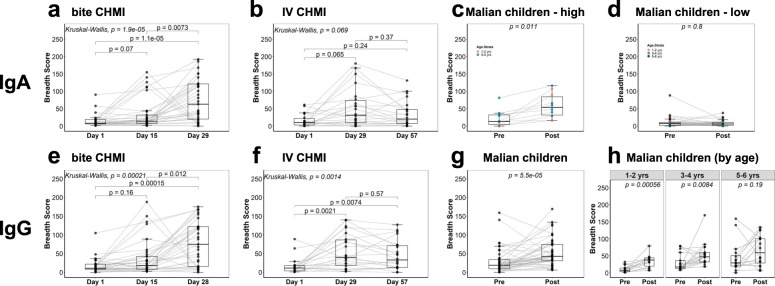
Change in number of *P. falciparum* proteins recognized by IgA and IgG for each individual (breadth score) in three clinical studies. Boxplots showing the comparisons of IgA and IgG breadth scores for bite CHMI (**a**, **e**), IV CHMI (**b**, **f**) and Malian children (**c**, **d**, **g**, **h**), over time demonstrate that breadth score increases after CHMI for both IgG and IgA. While IgG increases after infection in the Malian children (**g**), IgA increases after infection for the subset of the Malian children whose antibody responses had the highest correlation with CHMI adults (*n* = 9, **c**) but not for the remaining Malian children whose antibody responses had low correlation with CHMI adults (*n* = 38, **d**). IgG breadth scores for Malian children stratified by age (**h**) show greatest increase in breadth score for the youngest age stratum. Tests with only two groups used Wilcoxon sign rank test and comparisons of >2 groups used a Kruskal–Wallis test with corrected Dunn’s test *p* values.

The number of proteins recognized by IgG (breadth score) increased for participants in both CHMI studies (pre-infection compared to day 29) and after malaria illness in Malian children (Fig. 5e–g). The earlier timepoint (day 15) for the bite CHMI had breadth scores that were in between scores at the day 1 and day 29 timepoint (bite CHMI median breath scores for days 1, 15, and 29 were 11, 18, and 75, respectively; Kruskal Wallis *p* = 1.9 × 10^−5^). The breadth scores for the IV CHMI day 29 and 57 timepoints were similar (IV CHMI median breadth scores for days 1, 29, and 57 were 12, 40, and 33, respectively; day 1 vs. day 29 Wilcoxon *p* = 0.0021). For IgA, breadth scores increased after infection in the CHMI cohorts and in the subset of Malian children whose responses correlated with the adults who underwent CHMI, but not for the Malian children whose responses had low correlation with the CHMI adults (Fig. 5a–d). Overall, responses in all study groups were heterogeneous, with some individuals in each group having marked increases and others having no change in breadth score. For both IgG and IgA in the bite CHMI group, breadth scores were not statistically different when comparing groups that received different numbers of mosquito bites (1, 3, or 5 bites), although sample sizes were small (data not shown). For the IV CHMI study, breadth scores were similar for the low dose groups at baseline and post infection (*Kruskal Wallis p* = 0.51, IgA and *p* = 0.14, IgG) (Supplementary Fig. 6). Although some of the IgA breadth scores for the high dose IV CHMI group were higher than for the low dose group, results were not statistically significant (Kruskal Wallis *p* = 0.088, IgA) (Supplementary Fig. 6a). For IgG, the high dose group had a significant increase in breadth score after infection (*Kruskal Wallis p* = 0.011) (Supplementary Fig. 6b). Breadth scores were not statistically different when comparing the NF54 CHMI to the 7G8 CHMI, although the sample size was small (data not shown).

### Malian children IgG response correlated with age and time after infection

**Fig. 6:**
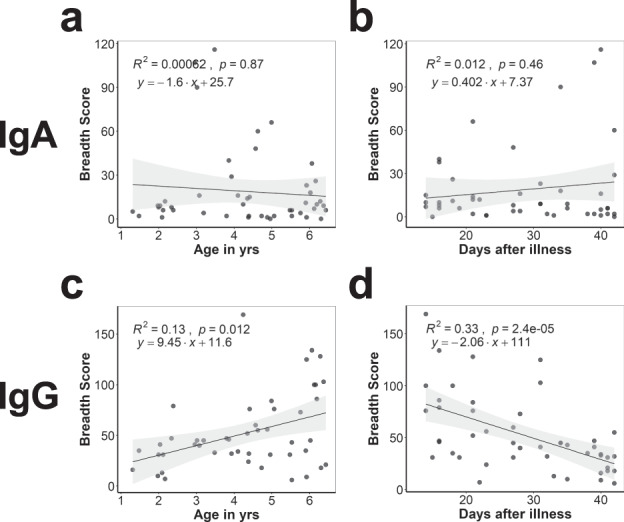
IgG response to *P. falciparum* antigens correlate with age and time after infection in Malian children. Relationship between change in number of *P. falciparum* proteins recognized by IgA and IgG for each individual (breadth score) and age at the time of post-infection blood draw (**a**, **c**) and days after infection (**b**, **d**) for Malian children are shown.

IgG breadth scores were increased in post- versus pre-infection samples (Fig. 5g, h). IgA breadth scores did not correlate with age or the number of days between illness and post-infection blood draw (Fig. 6a, b). However, IgG breadth scores were correlated with age (Spearman R^2^ = 0.13, *p* = 0.012, Fig. 6c) such that for each year of life from ages 1–6 years, the IgG breadth score increased by nine antigens. IgG breadth score had a negative correlation with the interval between illness and blood draw such that for each day after illness, the breadth score decreased by two antigens (Spearman R^2^ = 0.33, *p* = 0.000024, Fig. 6d), suggestive of short antibody half-life. Similar correlations were seen when looking at aggregate reactivity, defined as the sum of all isotype-specific fluorescence intensity (FI) for each individual (Supplementary Fig. 7).

## Discussion

This study compared antibody responses in children who had previous malaria exposures to antibody responses in adult volunteers who had a first malaria exposure by CHMI via two administration routes (mosquito bite and IV). We show that the serologic profile after natural infection in Malian children is different from the response to CHMI in malaria-naïve adults. Malian children demonstrated reduced IgA antibody prevalence and variability in the antigen specificity and breadth of IgG antibodies measured. These findings align with previously published results; healthy adult volunteers who underwent CHMI while simultaneously receiving chloroquine developed IgG responses that were specific to different *P. falciparum* proteins than those recognized by IgG responses in semi-immune Kenyan adults^11^. For the pediatric samples, both the increase in IgG responses with increasing age and the decrease in IgG responses with increasing days after infection for the post-infection blood draw demonstrate expected kinetics of IgG antibody responses in children^32,33^.

Lower IgA and IgG responses were observed in the IV CHMI study compared to the bite CHMI study (Supplementary Fig. 4). Ultra-sensitive PCR used to detect parasitemia in the IV CHMI study detects parasitemia 1–3 days sooner than thick blood smear used in the bite CHMI study. Because of this earlier detection of infection, the lower responses seen in the IV CHMI study could be due to earlier treatment that shortened exposure to parasite antigens. Lower IgA seroreactivity in Malian children may reflect their nascent immune systems, exposure to diverse rather than a single *P. falciparum* strain, or factors that lead to inhibition of antibody production^34^. Notably, when comparing the CHMI responses to responses in Malian children, we observed that CHMI elicited IgA to a greater breadth of antigens and with a higher intensity of seroreactivity than was observed in Malian children following natural infection. The production of IgA post-infection in malaria naïve individuals is an intriguing observation that has several possible explanations, which will be explored in planned future experiments, including experiments that test the extent to which IgA antibodies inhibit invasion of hepatocytes and/or erythrocytes.

One possible explanation for the observation of IgA following malaria infection is that mucosal immunity may be a significant but until recently under-recognized component of the immune response to *P. falciparum* malaria. In studies of a radiation-attenuated *P. falciparum* sporozoite vaccine (PfSPZ Vaccine) in Tanzanian adults^35^ and non-human primate hepatocytes^24^, mucosal-associated invariant T (MAIT) cells were observed following immunization. MAIT cells, a component of the innate immune response, are enriched at mucosal sites and have been observed to stimulate IgA, IgG, and IgM production in an ex vivo model^36^. Another study demonstrated that altered signal transduction and inflammation in host hepatocytes during the pre-erythrocytic phase of malaria infection results in upregulation of a human transmembrane mucin, MUC13^37^. The presence of MAIT cells and upregulation of a mucin both suggest a role of mucosal immunity in the response to *P. falciparum* infection, perhaps induced when pre-erythrocytic antigens traverse through the hepatic mucosal surface and elicit mucosal immune responses, including IgA. Alternatively, during malaria blood stage infection, γδ T cells migrate through colonic mucosa and subsequently induce an IgA response^38,39^. On the other hand, studies conducted in germ-free mice with no mucosal microbiota stimulus and in mice with defective intestinal homing signals demonstrate serum IgA production separate from mucosal IgA production^40–42^.

Our data are consistent with a recent report of experimental infection in naive mice with *Babesia*, a red blood cell pathogen that causes erythrocyte deformation and lysis, splenic enlargement, increase in number of splenocytes, and transient increases in IgA and IgG antibody titers within weeks^43^. In humans, malaria is associated with splenomegaly that may be provoked by the spleen’s main function of recycling aged and damaged red blood cells, including those that contain parasites^44^. Despite parasite-mediated changes in splenic architecture and resulting immunomodulation, the spleen mounts an immune response that includes production of antibodies from splenic plasma cells^45^. In humans, newly class-switched IgA circle transcripts were as abundant in the spleen as in gut mucosal sites and lymphoid follicles, indicating that the spleen is likely a major source of the predominating monomeric IgA1 in serum^46^. In mice, a recent review concludes that both peritoneal “innate-like” B1 B cells and splenic marginal zone B cells can contribute to IgA production^42^. Thus, our data suggest that a transient rise in newly generated IgA positive plasmablasts and plasma cells, possibly induced in the spleen, liver, gastrointestinal tract, and/or peritoneum of infected patients, may have given rise to the transient IgA titers seen in our study population.

A possible explanation for the IgA responses observed in only a subset of malaria-exposed children could include immune dysregulation in children. Chronic exposure to malaria leads to attenuation of the antibody response^34,47^. Bone marrow infection blunts the ability to mount B cell responses including diminution of B memory cells^48^ that could ultimately attenuate IgA production. In a mouse model, blood stage infection attenuated the development of antibodies to circumsporozoite protein, a highly immunogenic pre-erythrocytic antigen^49^. The method of IgA and IgG detection used in this microarray study precludes quantitative comparison of IgA to IgG, namely, each isotype was detected with a different fluorophore, and the fluorescence intensity rather than the amount of antibody was the readout. Thus, immune dysregulation in the setting of chronic infection may be more readily detectable as attenuated IgA levels rather than attenuated IgG levels.

Differential IgA responses in naïve adults vs. malaria-exposed children could alternatively or additionally be explained by timing-dependent changes in class switch recombination. In a human dengue virus challenge model, class-switched IgA plasmablasts following primary but not subsequent dengue infection were unexpectedly observed^50^. Likewise for malaria infections, the first parasite exposure may elicit an IgA response; on subsequent exposure an IgG dominant response may develop. For both dengue and malaria, controlled human infection models allow for the study of first infections in a naïve host. Even the youngest Malian children likely experienced a first exposure as infants while being protected by maternal antibodies; hence “first infections” may not have been observed in this Malian pediatric population and may explain the lower IgA response^33,34^. Previously, *P. falciparum* specific IgA was found in sera from malaria naïve participants undergoing bite-CHMI but not in semi-immune Kenyan adults^51^. IgA may not have been present in adult Kenyan serum because IgA may have a short half-life or because IgA is only elicited after first or early *P. falciparum* infections. Concomitantly, IgA levels have been shown to be higher in people who experienced fewer than five malaria infections^26^.

Post-infection IgA responses for a subset of the Malian children correlated with those of the adult CHMI participants, notably the children with more antibody responses above the detectable level (Fig. 2a, Supplementary Fig. 1). By contrast, IgG responses between Malian children and CHMI participants had low correlation. This observation suggests that, when considering infections that actually elicit IgA responses, Malian children and adult CHMI participants have similar IgA immunoproteomes. By contrast, the same two groups have distinct IgG immunoproteomes. Whereas the IgG immunoproteome represents antibody responses to pre-erythrocytic antigens for the adult CHMI participants and responses to both pre-erythrocytic and blood-stage antigens for the Malian children, the shared IgA immunoproteome may represent antibody responses to only pre-erythrocytic antigens. If postulated sites for development of IgA include liver, spleen, colon, and systemic circulation, perhaps the observation of a shared IgA immunoproteome suggests that IgA antibody responses develop at the site of the liver.

While all samples were probed on an array with proteins from the 3D7 strain, an important difference between the studies is the infecting parasite strain. The bite CHMI study used NF54, the parent strain of the 3D7 clone that is nearly genetically identical^52^. In the IV CHMI study, volunteers received either NF54 or 7G8, a Brazilian strain that was intentionally chosen because of its genetic distance from NF54. Naturally acquired malaria infections are from diverse parasites rather than specific strains. Therefore, it is possible that the detected antibody responses were lower in people who were infected with strains other than NF54. However, for the IV CHMI study, when serological responses to NF54 (*n* = 5) and 7G8 (*n* = 17) were compared, breadth score and seroreactivity did not differ between the two groups, albeit the sample size was small. Therefore, it is possible that strain specific humoral immune responses are difficult to see on a whole protein microarray because conserved immunodominant epitopes elicit high antibody binding^53^. Peptide microarrays may allow better discernment of strain-specific antibody responses.

Another important difference between malaria infection via CHMI vs. natural infection is the sporozoite dose. A dose of 3200 *P. falciparum* sporozoites (PfSPZ) or five infectious mosquito bites are standard for CHMI; however, these doses may be higher than what is experienced in any single natural infection. Moreover, the bite CHMI used aseptically reared mosquitos that have increased salivary gland PfSPZ counts as compared to conventionally reared mosquitoes, which may result in elevated challenge sporozoite doses. However, for the original aseptic mosquito bite CHMI study, although a trend was present for dose response when comparing number of bites to parasite density at time of malaria diagnosis^28^, in the current study we did not observe a dose response when comparing number of bites to IgA or IgG responses, suggesting that the sporozoite dose may not explain the increased antibody responses in CHMI compared to natural infection.

This study is significant because it compared the antibody response profile to malaria infections induced by mosquito bite vs. administration of aseptic, purified, cryopreserved PfSPZ administered intravenously by direct venous inoculation (PfSPZ Challenge), showing similarities and differences. Intravenous administration of PfSPZ Challenge is a practical way to conduct CHMI studies worldwide. Mosquito bite challenge, which requires insectaries for propagating infectious mosquitoes, is only available in a handful of institutions worldwide. Knowledge gained from the thorough analysis of these data will be valuable to interpret CHMI study results. This capacity will be important to malaria vaccine researchers and the greater vaccine research community.

The relevance of this work to malaria vaccine research and development is the expanded list of pre-erythrocytic antigens recognized by the immune system early post-infection and are candidates for a multi-antigen pre-erythrocytic vaccine. A vaccine against these target antigens would potentially induce sterile protective immunity and prevent malaria transmission.

## Methods

### Study participants

Serum samples from three previously conducted clinical trials were studied (Table 1):

#### Aseptic mosquito bite CHMI study

Malaria-naïve adults (*n* = 32; volunteers who had previously agreed to future use of their samples) aged 18–40 years received 1, 3, or 5 bites from aseptically reared *Anopheles stephensi* mosquitoes infected with the PfNF54 strain using Sanaria® PfSPZ Challenge Mosquitoes (ClinicalTrials.gov Identifier: NCT00744133)^28,29^. *P. falciparum* parasitemia was detected by thick blood smear and treated with chloroquine. Serum samples were collected before challenge and two- and four- weeks following challenge.

#### 7G8/NF54 intravenous PfSPZ challenge CHMI study

Malaria-naïve adults (*n* = 30) 18–45 years of age were randomly assigned to one of five treatment groups, four dose groups of Sanaria^®^ PfSPZ Challenge (7G8) or one dose group of PfSPZ Challenge (NF54), all administered intravenously (IV) by direct venous inoculation (ClinicalTrials.gov Identifier: NCT02780154)^30^. PfSPZ Challenge (7G8 and NF54) were composed of aseptic, purified, cryopreserved *P. falciparum* sporozoites (PfSPZ). Serum samples were collected before challenge, 29 and 56 days after challenge. *P. falciparum* parasitemia was detected by ultra-sensitive PCR in 22 participants and was treated with atovaquone/proguanil. Data from the 22 malaria infected participants were included in the final analysis.

#### Malian pediatric vaccine study

Malian children (*n* = 47) 1–6 years of age were randomized to receive an adjuvanted malaria subunit vaccine (AMA1/AS02A) or rabies vaccine as a control (ClinicalTrials.gov Identifier: NCT00460525)^31^. Only volunteers who received the control vaccine were included in the current study. From six scheduled blood draws throughout the first six months of the study, paired serum samples were selected for which a single intervening malaria illness occurred 6–41 days after the first blood draw and 14–42 days before the second blood draw. Malaria diagnosis was via thick blood smear, and all children received standard animalarial treatment when they presented with symptoms.

### Protein microarray methods

Protein microarrays displaying a total of 251 *P. falciparum* polypeptides representing 214 unique *P. falciparum* genes, derived from open reading frames from the Pf3D7 clone and down-selected from previous protein microarray studies based on immunogenicity were printed as in vitro transcription translation (IVTT) reactions and fabricated as previously described^54^. Seven control spots containing IVTT reaction substrates without DNA template were included on each array (“no-DNA” controls). Affinity-purified goat anti-human IgA and goat anti-human IgG conjugated to Qdot 585 and Qdot 800 (catalog numbers 110620 and 110630, respectively), were provided by Grace Bio-Labs, Inc., (Bend, OR). Serum samples were probed, arrays were imaged, and signals were quantified for IgA and IgG as previously described^51^. For each array, the mean of the fluorescence intensity (FI) of the no-DNA controls was subtracted from the FI of each of the other spots to create seroreactivity values for each sample-antigen pair. To minimize batch effect, all arrays were probed within two days. Samples from the three studies were randomized across all arrays with samples from the same individual but different timepoints probed on the same slides. Samples probed on different days and from different batches of slides were correlated, showing that slide printing and probing were reproducible. Samples from one participant were excluded from analysis because duplicate correlation was low.

### Statistical analysis

For timepoint comparisons, a baseline group that was comprised of Day 1 FI from both CHMI studies was compared to post-infection timepoints. A protein was considered differentially seroreactive for a study group when the median FI of the baseline group and the post-infection timepoint were significantly different by Wilcoxon signed-rank test. Proteins were considered differentially seroreactive between study groups when the median FI for the two study groups were significantly different by Wilcoxon rank sum test. For each study group, a two-component mixture model was constructed using seroreactivity for all proteins (*n* = 251), at all timepoints and all participants in the group. A cutoff point for each group was defined as the mean of the negative antibody signal distribution plus three standard deviations (SD) as previously described^10^. Antigens were defined as serorecognized by a sample if the seroreactivity was above the mixture model cutoff point. This approach allows for clustering observations (antibody responses) that are assumed to arise from a mixture of finite distributions (positive and negative antibody responses) against *P. falciparum* antigens on the array. Mixture models were created on a per-group basis with the presumption that pre-existing exposure was similar for each group. Seroprevalence was defined by study group for each antigen as the proportion of all samples with FI above the serorecognition threshold. Antigens with a seroprevalence of less than 10% amongst the post-infection samples in all three study groups were excluded from further analyses. The number of responders for each antigen per cohort was compared using a two-proportion *z* test with Yates continuity correction. All tests used *α* = 0.05 with application of the false discovery rate based on the Benjamini-Hochberg procedure. Boxplots are presented with median, interquartile ranges, and confidence interval defined as +/− 1.58*IQR/sqrt(n) indicated. All reported *p* values are two-sided. Analysis was conducted in R and visualized using ‘ggplot2’^55,56^. Venn diagrams for Supplementary Fig. 2 were constructed using http://bioinformatics.psb.ugent.be/webtools/Venn/.

### Ethics statement

Protocols were approved by institutional review boards of the University of Maryland, Baltimore (three clinical trial protocols) and the Faculty of Medicine, Pharmacy and Dentistry, Bamako, Mali (Malian pediatric vaccine study). Written informed consent was obtained from the participants of the Aseptic Mosquito Bite CHMI study, the IV CHMI study, and the unexposed controls. Written informed consent was obtained from the parents or guardians for the Malian pediatric vaccine study. All methods were performed in accordance with the relevant guidelines and regulations.

### Reporting summary

Further information on research design is available in the Nature Research Reporting Summary linked to this article.

## Supplementary information

Supplementary Information

Supplementary Data 1

Reporting Summary

## Data Availability

The data supporting the findings of this study are present within the article and the supplementary material, or are available from the authors upon request.
