# Supplementary material for: Immunoprofiles associated with controlled human malaria infection and naturally acquired immunity identify a shared IgA pre-erythrocytic immunoproteome
Source: NPJ Vaccines. 2021 Sep 13;6:115. doi: 10.1038/s41541-021-00363-y (PMC8438027; doi:10.1038/s41541-021-00363-y)
Supplement: Supplementary file 1 — Supplementary Information [file 41541_2021_363_MOESM1_ESM.pdf]

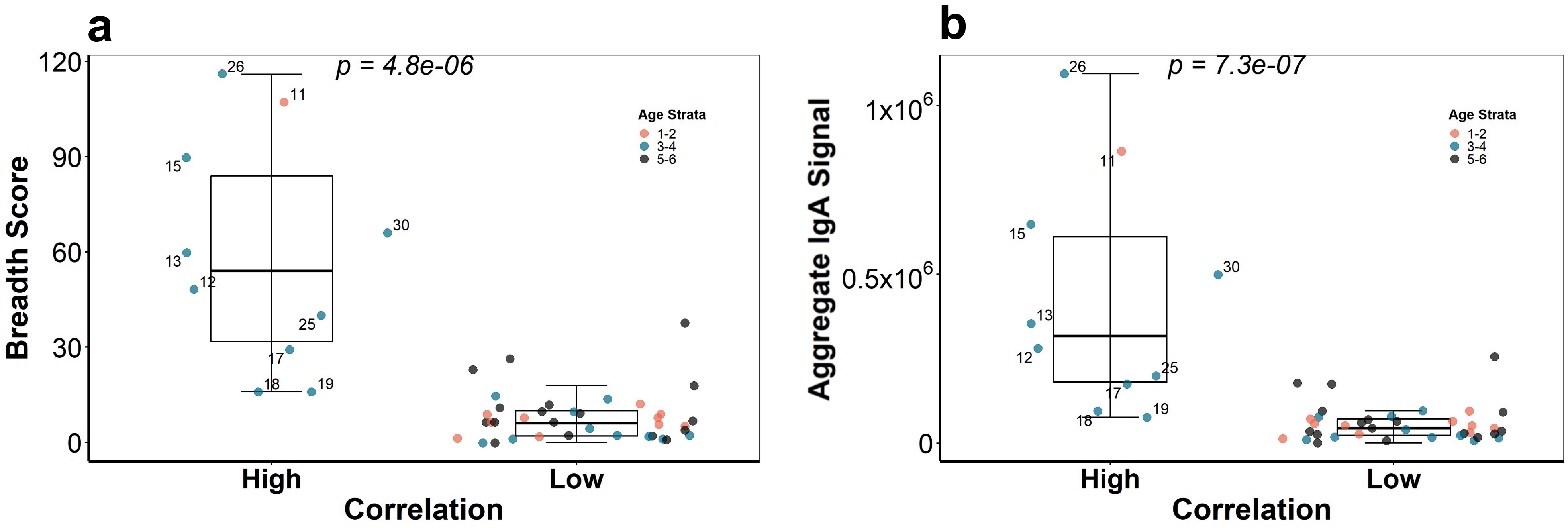

Supplementary Fig. 1 Relationship between IgA antibody responses to *P. falciparum* proteins in Malian children and correlation of responses with adult CHMI responses. High correlation boxplots represent ten post-infection samples with the highest correlation to adult CHMI samples (Fig. 2a, panels 4-5); low correlation boxplots represent the remaining samples, which had low correlation to adult CHMI samples (n = 37). Label numbers correspond with participant numbers in Fig. 2. Median breadth scores and aggregate IgA signal was higher for the samples with high correlation to adult CHMI samples as assessed by Wilcoxon-rank sum test. Breadth score is the change in number of *P. falciparum* proteins recognized by IgA for each individual. Aggregate seroreactivity is the sum of all IgA fluorescence intensity values for antigens identified as reactive.

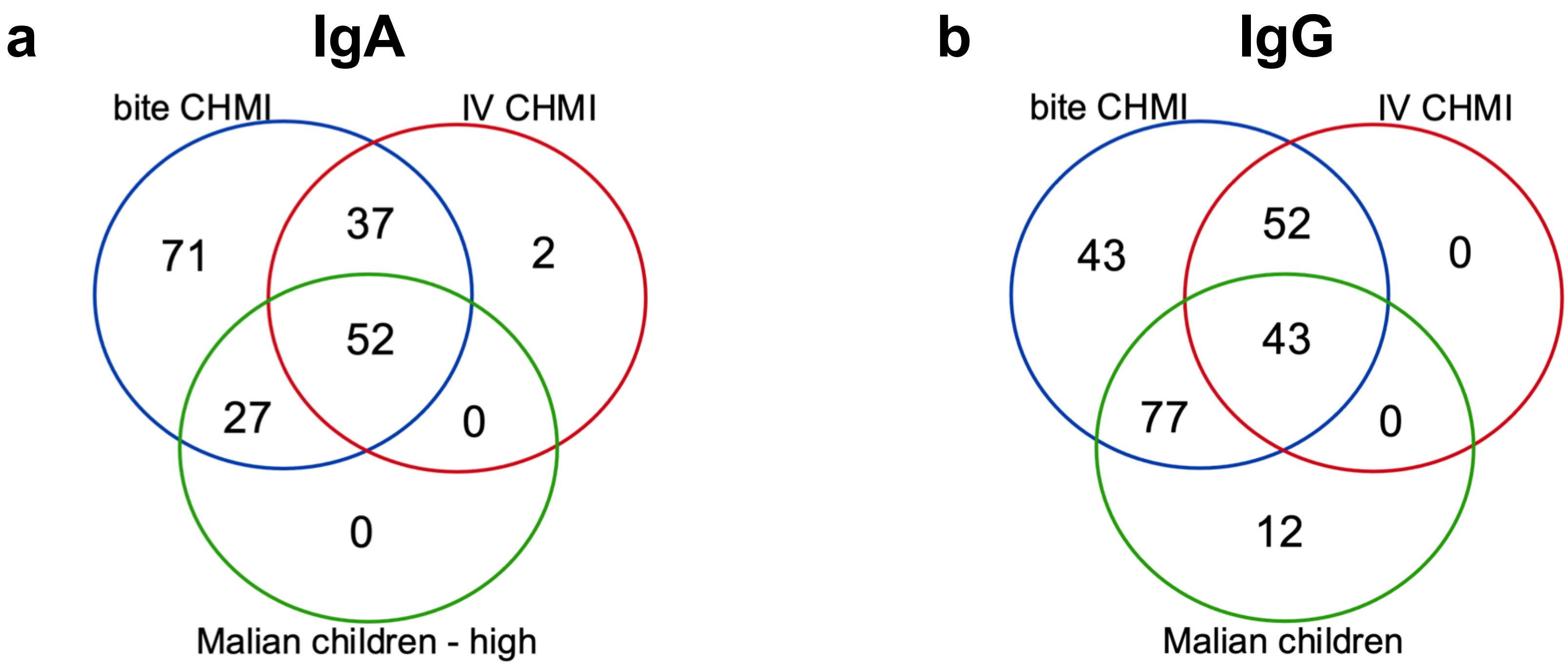

Supplementary Fig. 2 Venn diagrams displaying overlap of antigens for which IgA (a) and IgG (b) antibody reactivity to *P. falciparum* proteins were observed among the three study groups. For IgA, the subset of Malian children with antibody responses that were highly correlated with the CHMI participants (n=9) are shown.

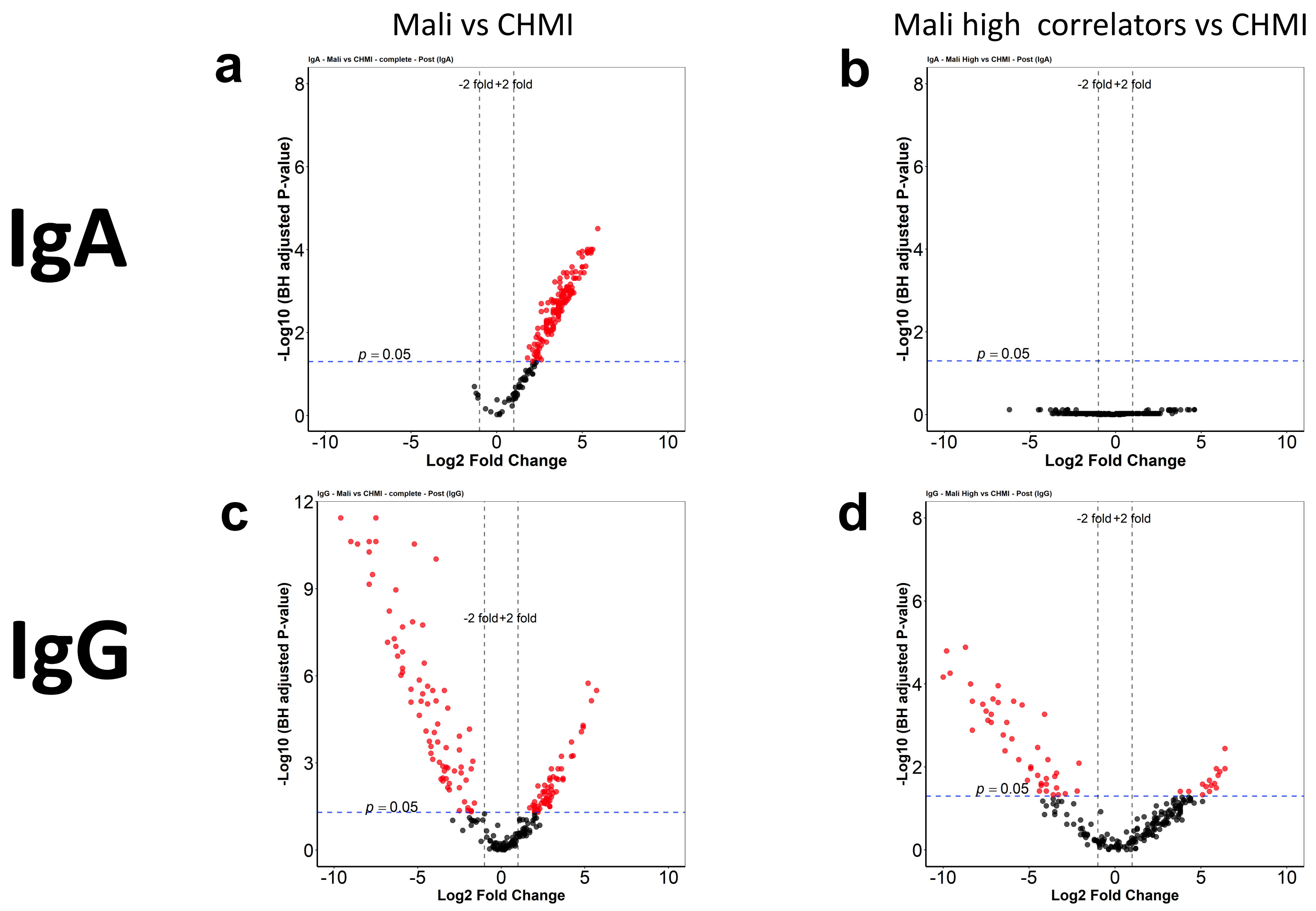

Supplementary Fig. 3. Comparison of IgA and IgG seroreactivity to *P. falciparum* proteins for Malian children vs. both CHMI cohorts. Volcano plots for IgA (a,b) and IgG (c,d) comparing post-infection signal intensities for each protein for Malian children (left of each plot) to the CHMI adults (right of each plot). CHMI groups have higher IgA seroreactivity compared to the Malian children (a). By contrast, the immunoproteomes of the CHMI groups and a subset of Malian children are simliar (b; highly correlated Malian children as illustrated in Fig 2). IgG reactivity is similar for Malian children vs. the highly correlated Malian children (d). Antigens are colored red if they pass the thresholds for false discovery rate (FDR) and Log Fold change. Antigens above the blue dashed line have Wilcoxon rank sum *p*-values < 0.05 after correction using the Benjamini Hochberg procedure. Analysis of 193 antigens for IgA and 228 antigens for IgG.

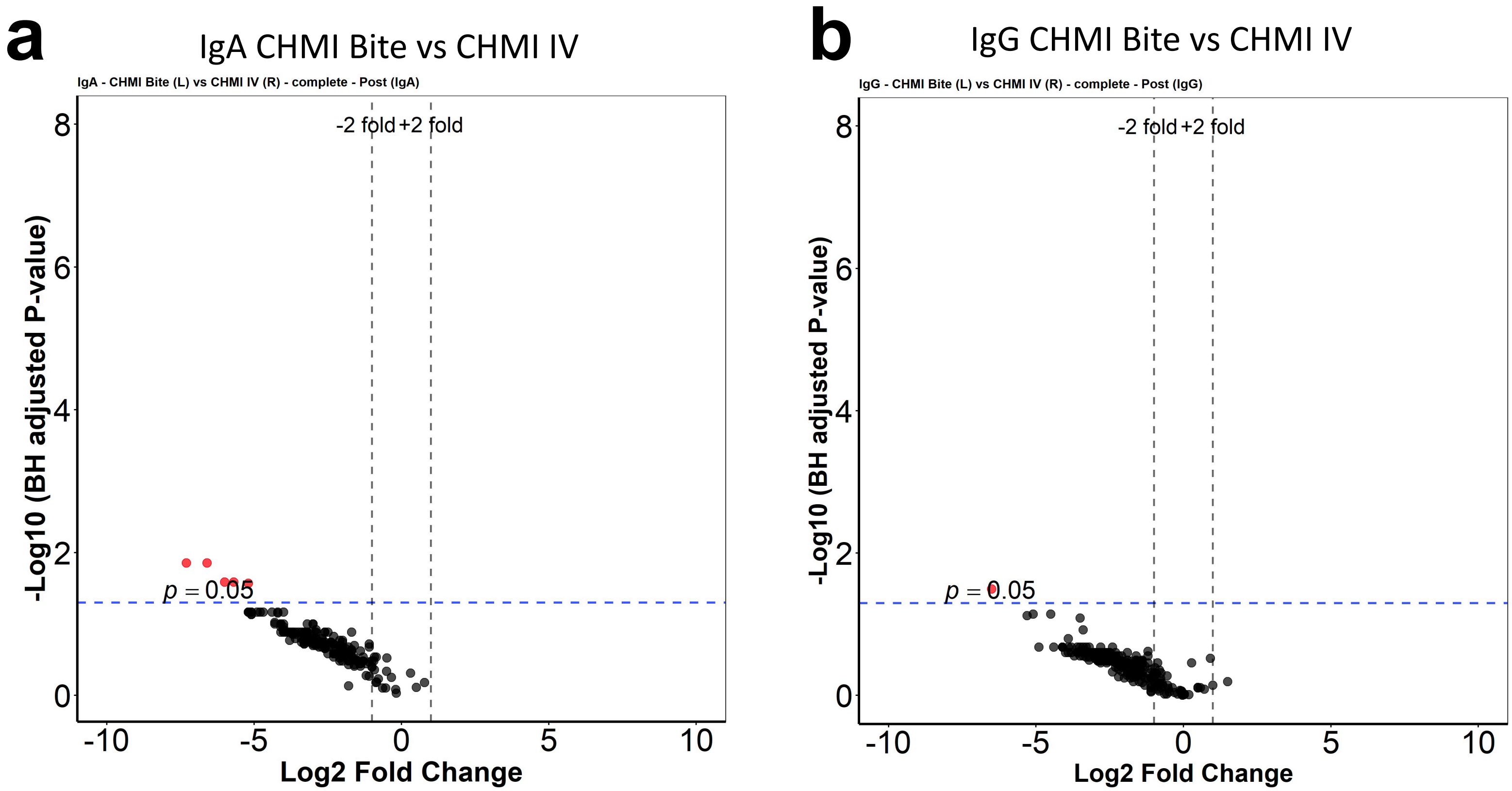

Supplementary Fig. 4. Comparison of IgA and IgG seroreactivity to *P. falciparum* proteins for the bite CHMI vs. IV CHMI groups. Volcano plots for IgA (a) and IgG (b) comparing post-infection signal intensities. For both IgA and IgG, seroreactivity is stronger for the bite CHMI group, but overall the immunoproteomes are similar for both groups. Analysis of 193 antigens for IgA and 228 antigens for IgG.

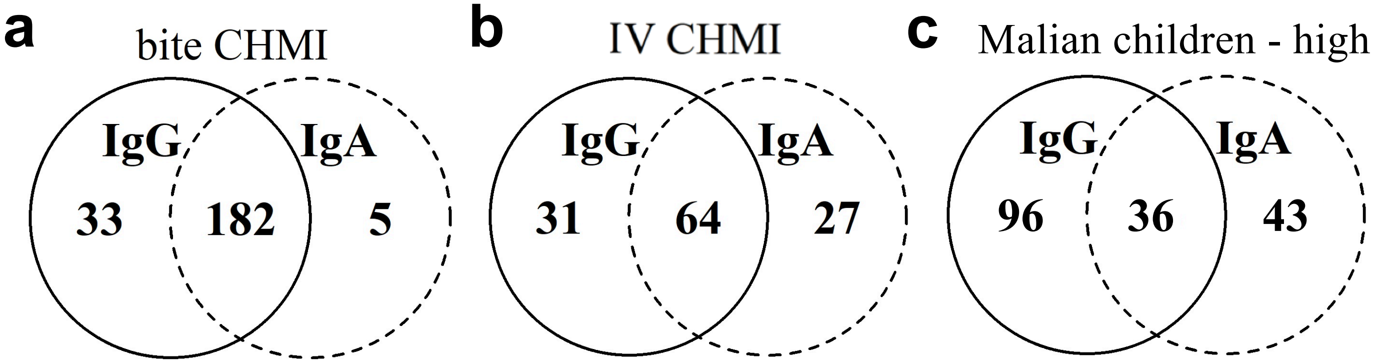

Supplementary Fig. 5 Venn diagrams displaying overlap of *P. falciparum* antigens that induced reactivity for both IgA and IgG for bite CHMI (a), intravenous (IV) CHMI (b) and the subset of Malian children with antibody responses that were highly correlated with the CHMI participants (c).

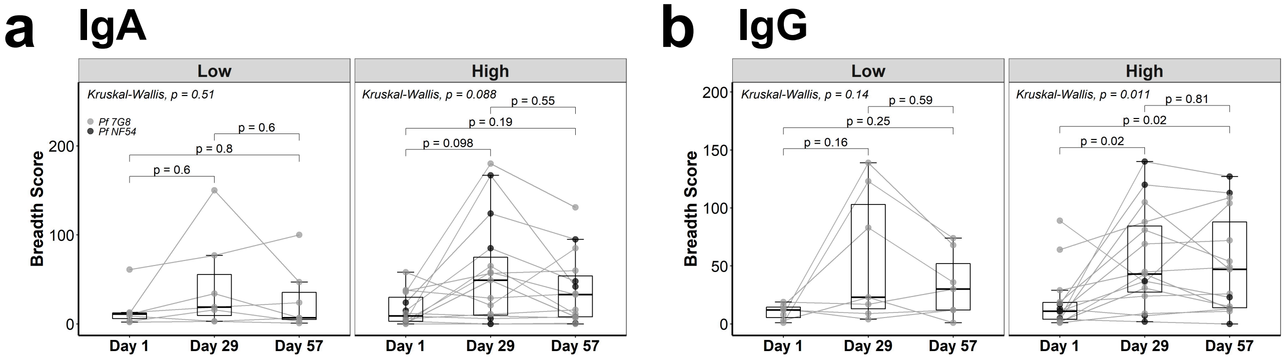

Supplementary Fig. 6 Change in number of *P. falciparum* proteins recognized by IgA and IgG for each individual (breadth score) in the 22 participants of intravenous (IV) CHMI study who developed parasitemia. Seven subjects received 800 or 1600 sporozoites (Low dose) and fifteen subjects received 3200 or 4800 sporozoites (High dose). Breadth scores for IgA (a) and IgG (b) are shown. Kruskal-Wallis test was used for comparisons of more than 2 groups and multiple comparisons were done using Dunn's test with *p-values* adjusted using the Benjamini-Hochberg procedure.

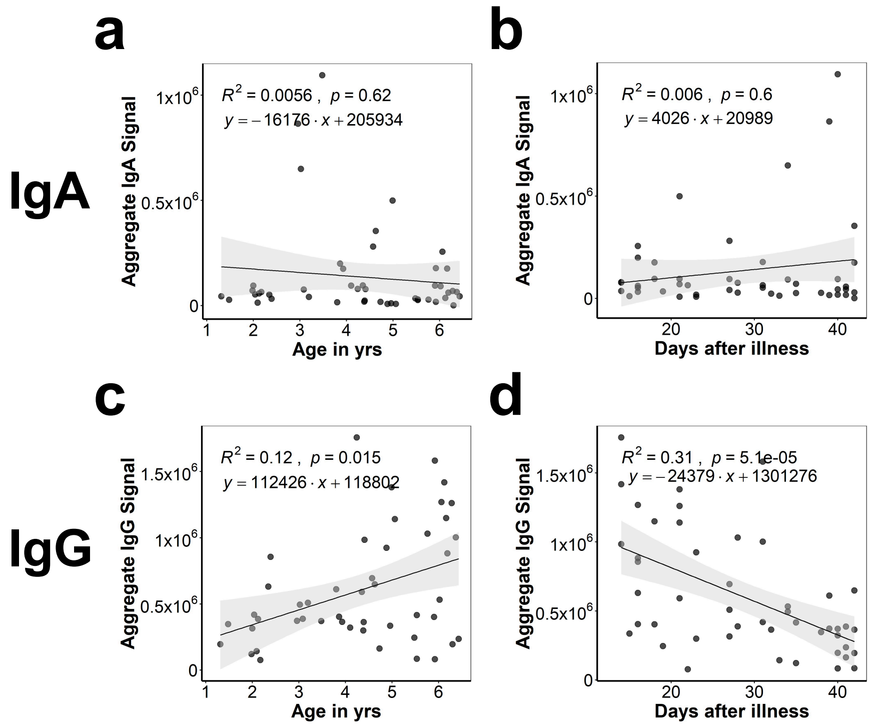

Supplementary Fig. 7 IgG aggregate antibody levels to *P. falciparum* proteins correlate with age and time after infection in Malian children. Relationship between aggregate antibody levels and age at the time of post-infection blood draw (a, c) and number of days after infection blood was drawn (b, d) for Malian children are shown. Aggregate antibody level is the sum of all isotype-specific fluorescence intensity (FI) values for antigens identified as reactive for each individual.

		Gene.ID	Name	Unique.ID	Log2 Fc	Adjusted p value
**Bite CHMI**						
	1	PF3D7_0206800	merozoite surface protein 2	PFB0300c	7.8	1.20E-07
	2	PF3D7_0823300	histone acetyltransferase GCN5	PF08_0034e1s1	7.7	1.30E-07
	3	PF3D7_0935900	ring-exported protein 1	PFI1735ce2s1	7.6	1.20E-07
	4	PF3D7_1036400	liver stage antigen 1	PF10_0356.1o2	7.3	2.20E-06
	5	PF3D7_0716300	conserved Plasmodium protein, unknown function	MAL7P1.77e1s1	7.2	4.40E-07
**IV CHMI**						
	1	PF3D7_0714500	transcription elongation factor s-II, putative	PF07_0057.1o1	5.1	0.0069
	2	PF3D7_0713900	conserved Plasmodium protein, unknown function	PF07_0053e1s3	4.9	0.0069
	3	PF3D7_1215900	serpentine receptor, putative	PFL0765w.1o1	4.7	0.0069
	4	PF3D7_0716300	conserved Plasmodium protein, unknown function	MAL7P1.77e1s1	4.6	0.0069
	5	PF3D7_1023100	dynein heavy chain, putative	PF10_0224e1s4	4.5	0.011
	6	PF3D7_0612600	cytoplasmic tRNA 2-thiolation protein 1, putative	PFF0610c.1o1	4.5	0.011
	7	PF3D7_1023100	dynein heavy chain, putative	PF14_0201	4.5	0.0069
**Mali Children**						
	1	PF3D7_0935900	ring-exported protein 1	PFI1735ce2s1	5.2	0.00036
	2	PF3D7_0206800	merozoite surface protein 2	PFB0300c	4.1	0.01
	3	PF3D7_0220000	liver stage antigen 3	PFB0915w-e2s1	3.2	0.019
	4	PF3D7_1035900	probable protein, unknown function	PF10_0351.1o1	2.8	0.01
**Mali high**						
	1	PF3D7_0826100	E3 ubiquitin-protein ligase, putative	MAL8P1.23-s8	9.1	1.80E-05
	2	PF3D7_0935900	ring-exported protein 1	PFI1735ce2s1	7.8	0.00044
	3	PF3D7_0323800	conserved Plasmodium protein, unknown function	PFC1065we2s1	7.7	1.30E-05
	4	PF3D7_1018300	conserved Plasmodium protein, unknown function	PF10_0177b	7.6	0.0011
	5	PF3D7_1433500	DNA topoisomerase 2	PF14_0316e1s1	7.4	0.0016

Supplementary Table 1. Top five P. falciparum antigens by IgA immunogenicity for three clinical studies.

	Gene.ID	Name	Unique.ID	Log2 Fc	Adjusted p value
	**Bite CHMI**				
1	PF3D7_0823300	histone acetyltransferase GCN5	PF08_0034e1s1	8.3	2.40E-09
2	PF3D7_0906200	conserved Plasmodium protein, unknown function	PFI0305c.1o1	7.6	1.80E-08
3	PF3D7_0935900	ring-exported protein 1	PFI1735ce2s1	7.5	4.30E-08
4	PF3D7_0714500	transcription elongation factor s-II, putative	PFI1735ce2s2	7.4	2.50E-08
5	PF3D7_0716300	conserved Plasmodium protein, unknown function	PFI1735ce2s3	7.3	1.60E-07
	**IV CHMI**				
1	PF3D7_0823300	histone acetyltransferase GCN5	PF08_0034e1s1	5.1	0.0052
2	PF3D7_0906200	conserved Plasmodium protein, unknown function	PFI0305c.1o1	4.9	0.0052
3	PF3D7_1300100	erythrocyte membrane protein 1, PfEMP1	MAL13P1.178-e2	4.9	0.0074
4	PF3D7_0714500	transcription elongation factor s-II, putative	PF07_0057.1o1	4.7	0.011
5	PF3D7_0612600	cytoplasmic tRNA 2-thiolation protein 1, putative	PFF0610c.1o1	4.4	0.011
6	PF3D7_1436300	translocon component PTEX150	PF14_0344e1s1	4.4	0.013
	**Mali Children**				
1	PF3D7_0935900	ring-exported protein 1	PFI1735ce2s1	12	6.60E-17
2	PF3D7_0220000	liver stage antigen 3	PFB0915w-e2s1	9.9	9.40E-14
3	PF3D7_0713900	conserved Plasmodium protein, unknown function	PF07_0053	9.5	3.60E-14
4	PF3D7_0801000	Plasmodium exported protein (PHISTc), unknown function	PF08_0137e2s2	9.1	1.80E-14
5	PF3D7_0801000	Plasmodium exported protein (PHISTc), unknown function	PF08_0137e2s1	9	6.50E-14

Supplementary Table 2. Top five P. falciparum antigens by IgG immunogenicity for three clinical studies
