# Supplementary material for: Immunoprofiles associated with controlled human malaria infection and naturally acquired immunity identify a shared IgA pre-erythrocytic immunoproteome
Source: NPJ Vaccines. 2021 Sep 13;6:115. doi: 10.1038/s41541-021-00363-y (PMC8438027; doi:10.1038/s41541-021-00363-y)
Supplement: Supplementary file 3 — Reporting Summary [file 41541_2021_363_MOESM3_ESM.pdf]

nature research

Corresponding author(s): Andrea A. Berry

Last updated by author(s): Jun 15, 2021

Reporting Summary

Nature Research wishes to improve the reproducibility of the work that we publish. This form provides structure for consistency and transparency in reporting. For further information on Nature Research policies, see our Editorial Policies and the Editorial Policy Checklist.

Statistics

For all statistical analyses, confirm that the following items are present in the figure legend, table legend, main text, or Methods section.

n/a	Confirmed	
☐	☒	The exact sample size (*n*) for each experimental group/condition, given as a discrete number and unit of measurement
☒	☐	A statement on whether measurements were taken from distinct samples or whether the same sample was measured repeatedly
☐	☒	The statistical test(s) used AND whether they are one- or two-sided<br>*Only common tests should be described solely by name; describe more complex techniques in the Methods section.*
☐	☒	A description of all covariates tested
☐	☒	A description of any assumptions or corrections, such as tests of normality and adjustment for multiple comparisons
☒	☐	A full description of the statistical parameters including central tendency (e.g. means) or other basic estimates (e.g. regression coefficient) AND variation (e.g. standard deviation) or associated estimates of uncertainty (e.g. confidence intervals)
☒	☐	For null hypothesis testing, the test statistic (e.g. *F*, *t*, *r*) with confidence intervals, effect sizes, degrees of freedom and *P* value noted<br>*Give P values as exact values whenever suitable.*
☒	☐	For Bayesian analysis, information on the choice of priors and Markov chain Monte Carlo settings
☒	☐	For hierarchical and complex designs, identification of the appropriate level for tests and full reporting of outcomes
☒	☐	Estimates of effect sizes (e.g. Cohen's *d*, Pearson's *r*), indicating how they were calculated

Our web collection on statistics for biologists contains articles on many of the points above.

Software and code

Policy information about availability of computer code

Data collection	Data was collected from the Grace Bio-Labs imager as previously described and assembled using a custom Microsoft Excel Macro that is available upon request.
Data analysis	Analysis was conducted in R and visualized using 'ggplot2'. Venn diagrams were constructed using http://bioinformatics.psb.ugent.be/webtools/Venn/ .

For manuscripts utilizing custom algorithms or software that are central to the research but not yet described in published literature, software must be made available to editors and reviewers. We strongly encourage code deposition in a community repository (e.g. GitHub). See the Nature Research guidelines for submitting code & software for further information.

Data

Policy information about availability of data

All manuscripts must include a data availability statement. This statement should provide the following information, where applicable:

- Accession codes, unique identifiers, or web links for publicly available datasets
- A list of figures that have associated raw data
- A description of any restrictions on data availability

The data supporting the findings of this study are present within the article and the supplementary material, or are available from the authors upon request.

Field-specific reporting

Please select the one below that is the best fit for your research. If you are not sure, read the appropriate sections before making your selection.

☒ Life sciences ☐ Behavioural & social sciences ☐ Ecological, evolutionary & environmental sciences

For a reference copy of the document with all sections, see nature.com/documents/nr-reporting-summary-flat.pdf

Life sciences study design

All studies must disclose on these points even when the disclosure is negative.

Sample size	32 bite CHMI participants; 33 IV CHMI participants; 47 Malian children
Data exclusions	A sample was excluded from the Malian set because duplicate correlation was low.
Replication	Some samples were run in duplicate. Analysis was done in R.
Randomization	Pre- and post- infection samples were chosen from all available samples of the 2 CHMI studies. Malian samples were chosen from a larger study as follows: Malian children (n = 47) 1-6 years of age were randomized to receive an adjuvanted malaria subunit vaccine (AMA1/AS02A) or rabies vaccine as a control.31 Only volunteers who received the control vaccine were included in the current study. From six scheduled blood draws throughout the first six months of the study, paired serum samples were selected for which a single intervening malaria illness occurred at least 6-41 days after the first blood draw and 14-42 days before the second blood draw.
Blinding	The team members who probed the microarrays did not have metadata or information about the samples being run at the time that the experiments were performed.

Reporting for specific materials, systems and methods

We require information from authors about some types of materials, experimental systems and methods used in many studies. Here, indicate whether each material, system or method listed is relevant to your study. If you are not sure if a list item applies to your research, read the appropriate section before selecting a response.

Materials & experimental systems

n/a	Involved in the study	
☐	☒	Antibodies
☒	☐	Eukaryotic cell lines
☒	☐	Palaeontology and archaeology
☒	☐	Animals and other organisms
☐	☒	Human research participants
☐	☒	Clinical data
☒	☐	Dual use research of concern

Methods

n/a	Involved in the study	
☒	☐	ChIP-seq
☒	☐	Flow cytometry
☒	☐	MRI-based neuroimaging

Antibodies

Antibodies used	Affinity-purified goat anti-human IgA and goat anti-human IgG conjugated to Qdot 585 and Qdot 800 (catalog numbers 110620 and 110630, respectively), were provided by Grace Bio-Labs, Inc., (Bend, OR).
Validation	Antibodies were previously validated (PMCID: PMC6637747) and afterwards validity is confirmed by comparing results from control samples that are run across all experiments over time.

Human research participants

Policy information about studies involving human research participants

Population characteristics	*Describe the covariate-relevant population characteristics of the human research participants (e.g. age, gender, genotypic information, past and current diagnosis and treatment categories). If you filled out the behavioural & social sciences study design questions and have nothing to add here, write "See above."*
Recruitment	*Describe how participants were recruited. Outline any potential self-selection bias or other biases that may be present and how these are likely to impact results.*
Ethics oversight	*Identify the organization(s) that approved the study protocol.*

Note that full information on the approval of the study protocol must also be provided in the manuscript.

Clinical data

Policy information about clinical studies
All manuscripts should comply with the ICMJE guidelines for publication of clinical research and a completed CONSORT checklist must be included with all submissions.

Clinical trial registration	*Provide the trial registration number from ClinicalTrials.gov or an equivalent agency.*
Study protocol	*Note where the full trial protocol can be accessed OR if not available, explain why.*
Data collection	*Describe the settings and locales of data collection, noting the time periods of recruitment and data collection.*
Outcomes	*Describe how you pre-defined primary and secondary outcome measures and how you assessed these measures.*
